# Emergence of mTOR mutation as an acquired resistance mechanism to AKT inhibition, and subsequent response to mTORC1/2 inhibition

**DOI:** 10.1038/s41698-021-00240-w

**Published:** 2021-12-01

**Authors:** Niamh Coleman, Vivek Subbiah, Shubham Pant, Keyur Patel, Sinchita Roy-Chowdhuri, Sireesha Yedururi, Amber Johnson, Timothy A. Yap, Jordi Rodon, Kenna Shaw, Funda Meric-Bernstam

**Affiliations:** 1 Department of Investigational Cancer Therapeutics (Phase I Program), The University of Texas MD Anderson Cancer Center, 1515 Holcombe Blvd, Houston, TX 77030 USA; 2 Khalifa Institute for Personalized Cancer Therapy, MD Anderson Cancer Center, Houston, TX USA; 3 Department of Pathology, MD Anderson Cancer Center, Houston, TX USA; 4 Abdominal Imaging Department, MD Anderson Cancer Center, Houston, TX USA; 5 Department of Surgical Oncology, MD Anderson Cancer Center, Houston, TX USA

**Keywords:** Tumour biomarkers, Oncogenes

## Abstract

Acquired resistance to molecular targeted therapy is a significant challenge of the precision medicine era. The ability to understand these mechanisms of resistance may improve patient selection and allow for the development of rationally designed next-line or combination treatment strategies and improved patient outcomes. AKT is a critical effector of the phosphoinositide 3-kinase signaling cascade, one of the most commonly activated pathways in human cancer. Deregulation of signaling pathways, such as RAF/MEK/ERK are previously described mechanisms of resistance to AKT/PI3K inhibitors. Mutations in the mTOR gene, however, are exceedingly rare. We present a case of acquired mTOR resistance, following targeted AKT inhibition, and subsequent response to mTOR1/2 inhibitor in a patient with metastatic endometrial cancer, the first documented response to ATP-competitive mTOR inhibition in this setting. This case supports mTOR mutation as a mechanism of resistance, and underscores the importance of tumor molecular profiling, exemplifying precision medicine in action.

## Introduction

The serine/threonine kinase, AKT, is a critical effector of the phosphoinositide 3-kinase (PI3K) signaling cascade and is one of the most commonly activated pathways in human cancer^1^. Dysregulation of AKT-dependent pathways is associated with the development and maintenance of various solid tumors, such as those of the endometrium, cervix, lung, prostate, skin, and breast^2–4^. Thus, AKT remains an intensely pursued therapeutic target in the era of precision medicine. Indeed, there are a number of small-molecule inhibitors targeting various components of the PI3K/AKT pathway currently at various stages of clinical development, in multiple solid tumors, including prostate, gastric, and breast cancer^5–7^.

There are several AKT inhibitors in clinical development, which predominantly fall into two separate classes: ATP-competitive inhibitors of AKT, which bind to the active site of AKT, blocking ATP binding (e.g., ipatasertib and capivasertib) and allosteric inhibitors of the AKT PH-domain which prevent localization of AKT to the plasma membrane, thereby blocking AKT phosphorylation and activation (e.g., ARQ 751 (ArQule) and MK-2206). Capivasertib (AZD5363) is an oral, potent, selective ATP-competitive pan-AKT kinase inhibitor, which has demonstrated clinical activity in patients with heavily pretreated AKT1 E17K mutant solid tumors, with confirmed partial responses reported in ER- endometrial, breast, cervical, and lung cancer^8^. Furthermore, capivasertib demonstrated a 28.6% ORR in the National Cancer Institute MATCH subprotocol (EAY131-Y) in patients with AKT1 E17K mutant tumors^9^. Capivasertib plus fulvestrant has also shown antitumor activity in heavily pretreated patients with PTEN-mutated ER + metastatic breast cancer (MBC), including those with prior progression on fulvestrant^9^.

Ipatasertib (GDC-0068), another ATP-competitive pan-AKT kinase inhibitor, has also shown clinical activity in combination with fulvestrant in patients with AKT1 E17K mutant MBC^10,11^, and has been explored in this population. Although in a Phase III trial, ipatasertib in combination with paclitaxel has not enhanced PFS compared to paclitaxel in MBC, in the Phase III IPATential150 trial^12^, ipatasertib improved radiographic progression-free survival in metastatic castration-resistant prostate cancer (mCRPC) and patients whose tumors had PTEN loss^13^. Previously, we have shown that ARQ 751 demonstrated a manageable safety profile, and four patients achieved the best response of stable disease, including one with MBC treated for 42+ weeks; the dose escalation is currently ongoing^14^.

Acquired resistance is a major challenge for molecularly targeted therapies, and understanding these mechanisms of resistance may improve patient selection and allow the development of rationally designed next-line or combination treatment strategies. Here, we present a case of acquired mTOR resistance, following targeted AKT inhibition, and subsequent response to mTOR1/2 inhibitor in a patient with metastatic endometrial carcinoma. To our knowledge, this is the first documented case of mTOR mutation as an acquired mechanism of resistance in the setting of AKT inhibition, and subsequent documented response to mTOR-based targeted therapy.

## Results

### Case

**Fig. 1:**
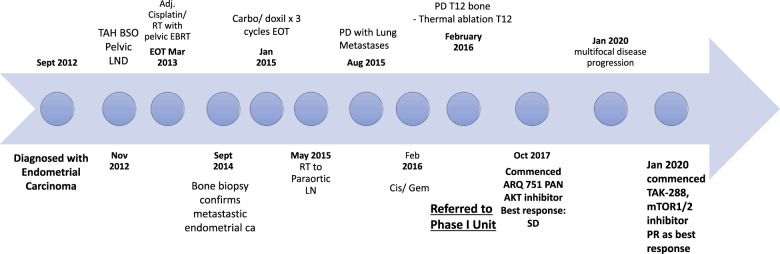
Treatment timeline. Timeline summarizing treatment course of the patient, including all systemic treatments that the patient received.

A 60-year-old female patient, with a prior history of breast cancer, presented with abnormal vaginal bleeding, and biopsy-confirmed grade II endometrial adenocarcinoma with squamous metaplasia. Following her diagnosis, she initially underwent total laparoscopic hysterectomy with pelvic/aortic lymph node dissection, and pathology confirmed IB grade II endometrial adenocarcinoma with squamous metaplasia, and lymphovascular space invasion. Following this, the patient received adjuvant cisplatin and radiotherapy, with pelvic external beam radiotherapy and vaginal brachytherapy (Fig. 1).

One year later, she developed persistent back pain, and imaging revealed a single T12 lesion with epidural impingement, and subsequent tumor biopsy confirmed metastatic adenocarcinoma consistent with endometrial cancer. Systemic restaging studies revealed a small but suspicious left-sided para-aortic lymph node at the levels of L2-L3. The patient underwent stereotactic radiosurgery and thermal ablation for cord compression, and she received carboplatin and liposomal doxorubicin for three cycles, followed by focal radiotherapy to persistent para-aortic lymphadenopathy.

Computed tomography (CT) restaging imaging 7 months later revealed disease progression in the lungs, and the patient commenced cisplatin and gemcitabine chemotherapy. However, post cycle 3, imaging revealed T12 metastasis showing a new region of FDG-avidity, compatible with progression and stable pulmonary nodules. MRI spine showed suspected progression of epidural soft tissue metastasis at T12 resulting in mild-to-moderate spinal canal stenosis but no definite cord signal abnormality. She received definitive treatment for her cancer in the spine, with thermal ablation of the T12 region, followed by vertebrectomy and reconstruction with cement stabilization T9-L3, and zolendronic acid therapy was commenced.

Following the further progression of cancer on positron emission tomography (PET) CT, with the increase in size and number of pulmonary metastases and new mesenteric disease, the patient was referred to for consideration of a phase I trial Department of Investigational Cancer Therapeutics at the University of Texas MD Anderson Cancer Center. Next-generation sequencing (NGS)– analysis for the detection of somatic mutations in the coding sequence of 50 genes^15^ (Ion Ampliseq 50-Gene Assay; Thermo Fisher) of retroperitoneal lymph node revealed an AKT1 E17K activating mutation and no other co-occurring alterations of functional significance (Supplementary Table 1). The patient was enrolled on a phase I study of an allosteric pan-AKT inhibitor ARQ751 (NCT02761694). The patient received 25 mg daily orally initially and tolerated treatment without significant toxicity. As the protocol allowed intra-patient dose-escalation, the dose was escalated to 50 mg daily and subsequently increased to 75 mg daily. Treatment was relatively well-tolerated, requiring no dose reductions or interruptions of treatment. She experienced stability of disease (4% reduction by RECIST criteria version 1.1)^16^ lasting 27 months.

Following 27 months of AKT inhibition, RECISTv1.1 progressive disease was confirmed on restaging PET-CT imaging, showing an increase in the size of multifocal pulmonary metastases and increase in left axillary nodal metastatic disease. The patient underwent a CT-guided lung biopsy which confirmed adenocarcinoma consistent with the endometrial primary. NGS analysis (Oncomine®, Thermo Fisher) for the detection of somatic mutations in the coding sequence of 143 cancer-related genes^17^ was conducted, on this occasion on the DNA extracted from the patients’ metastatic pulmonary lesion, which confirmed the persistence of AKT1 E17K mutation, and an mTOR mutation, mTOR A1459D, annotated to be an activating mTOR mutation. Sequencing on the lung metastasis demonstrated a CTNNB1 G34R mutation which was also noted on the 50 gene panel of the initial sample. There were no other co-occurring alterations identified. Although the original 50 gene hotspot panel did not sequence mTOR, it was hypothesized that the mTOR mutation may be an acquired resistance mutation, thus, the original pre-treatment sample was re-sequenced on the Oncomine platform, confirming that the AKT1 E17K mutation, and but not the mTOR A1459D mutation was detected (Supplementary Table 1).

**Fig. 2:**
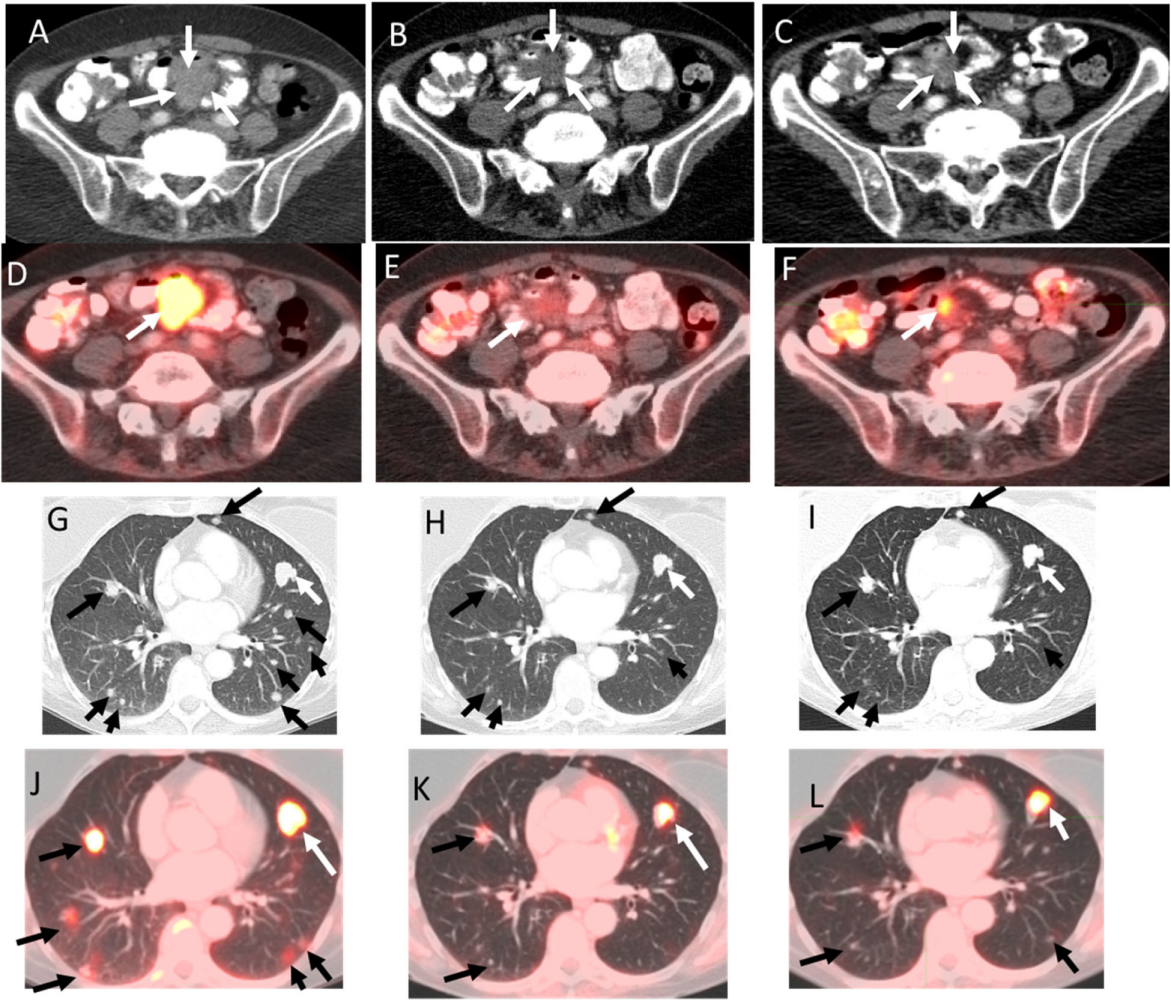
Serial axial CT and fused PET/CT images from contrast enhanced PET/CT examinations at baseline, 4.5 months and 8.5 months illustrate disease response. Serial axial CT (**A**–**C**) and fused PET/CT (**D**–**F**) images of the abdomen in soft tissue window show response in the mesenteric implant with decreased size (white arrows in **A**–**F**) and significantly decreased FDG uptake with a maximum SUV of 10.5 at baseline, 3.6 at 4.5 months follow up and 4.8 at 8.5 months follow up. Serial axial CT (**G-I**), and fused PET/CT (**J-L**) images in lung windows showed a response in the lung metastases. The largest metastasis in the lingua remained grossly stable in size (white arrows in **G-I**) but showed decreased uptake with a maximum SUV of 20.1 at baseline, 8.8 at 4.5 months follow up and 8.6 at 8.5 months follow up. Multiple other smaller lung metastases (black arrows in **G-L**) also showed decreased size and decreased uptake.

The patient was subsequently enrolled on a phase I study of the mTORC1/2 inhibitor sapanisertib (TAK-228) given 4 mg daily with metformin 500 mg twice daily (NCT03017833). The patient achieved a confirmed partial response (PR) by RECIST criteria version 1.1^16^ post-6 cycles of therapy (maximum reduction 30% from baseline) (Fig. 2). The patient remains on trial at 14 months with good tolerability.

## Discussion

AKT/PI3K/mTOR signaling is commonly disrupted in human cancers, with AKT being a central component of the pathway, influencing multiple processes which are directly involved in tumorigenesis. AKT is a family of serine/threonine kinases consisting of three isoforms (AKT1, AKT2, and AKT3), regulated upstream by the activation of PI3K, following growth factor stimulation. Several downstream substrates of activated AKT play a major role in the regulation of cell size, cell cycle progression, glucose metabolism, genome stability, transcription, protein synthesis, and inhibition of pro-apoptotic proteins^18–20^. Targeting this pathway has, therefore, been a highly attractive anti-cancer strategy and significant efforts have been made to target this kinase for many years.

Acquired resistance to molecular targeted therapy represents a significant challenge for the effective treatment of cancer. Deregulation of signaling pathways, including alterations in Raf/Mek/ERK are previously described determinants of tumor resistance to AKT/PI3K inhibitors^21,22^. We report, to the best of our knowledge, the first clinical case of acquired resistance following targeted therapy with AKT inhibition due to the development of an activating mTOR mutation, and following subsequent detection of this lesion, the first clinical case of documented response to mTOR inhibition in this setting.

The AKT mutation described in our patient, E17K, is found in the PH domain of AKT1 where a glutamic acid is substituted with a lysine residue at amino acid 17 (E17K), results in enhanced activity of the kinase, leading to constitutive membrane localization of the kinase and increased phosphorylation on T308 and S473 in a PI3K-independent manner^23,24^. Upregulation of AKT3 has been suggested as a potential mechanism of resistance to allosteric AKT inhibitor MK2206, using preclinical breast cancer models^25^. Target engagement can, however, be significantly influenced by drug-specific and drug-class-specific differences in isoform and conformation selectivity, and also by the effects of mutation on the accessibility to drug binding sites. For instance, activating AKT mutations such as AKT1-E17K, can destabilize the PH-in conformation and therefore confer resistance to allosteric AKT inhibitors but sensitivity to ATP-competitive inhibitors^26^. Therefore, the mechanisms of resistance to different AKT inhibitors may be different.

mTOR is a serine-threonine kinase that forms two physically and functionally distinct protein signaling complexes, mTORC1 and mTORC2, which are distinct in their regulation, susceptibility to different classes of inhibitors, and downstream substrates^27^. Multiple independent studies in cellular and mouse models have demonstrated that sustained, or incompletely inhibited, mTORC1 signaling can contribute to TKI resistance in *EGFR-*mutant NSCLC and *BRAF*-mutant melanoma^28^. Moreover, pre-clinical data are implicating mTORC signaling in resistance to PI3K inhibitors: *PIK3CA*-mutant breast cancer models resistant to PI3K inhibitors have been shown to exhibit sustained mTORC1 signaling^29^, and treatment with a rapalog was sufficient to sensitize resistant cells to PI3Kα inhibitor alpelisib^29^. Indeed, activation of mTORC1 has been shown to be a key event in resistance to PI3K inhibitors in a number of tumors types, perhaps because of its role downstream of PI3K^28^. In addition to this, the concomitant inhibition of PI3K and mTORC1 has been proven to sensitize resistant cell lines in breast and head and neck cancer, suggesting that mTORC1 may play a role in limiting the sensitivity to PI3K^30^.

**Fig. 3:**
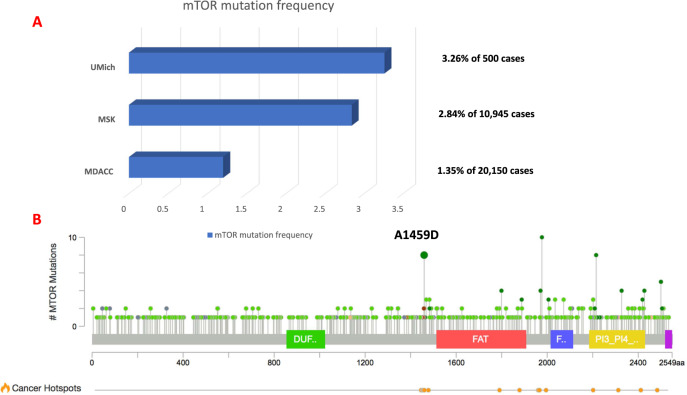
Frequency of mTOR mutations. **A** Interrogation of IPCT database in MDACC found that of 20,150 patients sequenced, 273 cases of mTOR mutation were identified; cbioportal was used to identify the frequency in other datasets, including Metastatic Solid Cancers (UMich, Nature 2017) and the MSK-IMPACT Clinical Sequencing Cohort (MSKCC, Nat Med 2017)^46,47^. **B** Lollipop plot showing the distribution of mTOR mutations, including the mTOR A1459D mutation, detected in this case^46,47^.

Mutations in the mTOR gene are rare, and on the interrogation of the Institute for Personalized Cancer Therapy (IPCT) database in MD Anderson Cancer Center, we noted a frequency of 1.35% of 20,150 patients screened (platforms included CMS400, STGAv1, STGA DNA2018, and LBPv1) (Fig. 3). The mTOR A1459D alteration found in our patient is located within the FAT domain of MTOR (amino acids 1382-1982, UniProt) and TPR repeat 4 (amino acids 1443-1473, UniProt) and has been reported as a recurrent mutation (Fig. 3). This alteration has been reported in a patient with hemimegalencephaly a disorder caused by mutations that result in activation of the PI3K pathway^31^. Analysis of resected brain tissue from the patient revealed hyper-phosphorylation of mTOR’s downstream targets. Additionally, another study identified this mutation in focal cortical dysplasia type IIb^32^. In this study, the researchers also observed increased phosphorylation of the mTOR target, 4EBP, compared with cells expressing wild-type mTOR. Another variation of this codon, A1459P, was experimentally shown to also confer a gain-of-function through reduced interaction with the negative regulator, DEPTOR^33^. Thus, we conclude that this alteration leads to a gain-of-function.

Some activating mTOR mutations are sensitive to rapamycin treatment^34^, and while genomic studies have linked mTORC1 pathway–activating mutations with exceptional response to treatment with rapalogs, preclinical studies have also shown that some mTOR mutations can increase mTORC2 activities^35,36^. Notably, Wagle et al. reported the emergence of an mTOR F2108L mutation in patient with anaplastic thyroid cancer bearing an inactivating TSC2 mutation, after 18 months of treatment on everolimus. Similarly, we have also preclinically identified an acquired resistance mutation in mTOR (mTOR S2035F) with continuous in vitro rapalog treatment and demonstrated that cancer cells with this mutation are resistant to everolimus but are still sensitive to mTOR catalytic inhibitor TAK228 in vitro and in vivo^37^. Altogether, these data suggest that mTOR mutations may indeed be a mechanism of resistance to allosteric mTOR inhibitors, with a potential role for catalytic inhibitors^38^. However, this mechanism of resistance is likely to be rare, and in Phase II trials sapanisertib showed only modest clinical benefit in breast cancer patients previously treated with everolimus^39^.

Cells expressing another variant of this codon, mTOR A1459P, were found to be still sensitive to mTOR inhibition with rapamycin^33^. Thus, it is unclear whether mTOR_A1459D selectively benefitted from catalytic mTOR inhibition or whether it would also have been sensitive to rapamycin analogs. In our previous preclinical work, we have shown that combination of allosteric AKT inhibitor MK2206 and allosteric mTOR inhibitor rapamycin are synergistic in vitro, with greater pathway inhibition as well as greater induction of apoptosis, and the combination leads to a greater enhancement of antitumor activity in vivo^40^. Thus, the combination of Akt/mTOR inhibition may be another strategy worth exploring to achieve deeper responses but may be limited due to tolerability concerns.

Our case report has a few limitations. Our patient had her tumor sequenced on two occasions using different metastatic disease sites (Supplementary Table 1), and NGS data-guided therapeutic strategy development. The first biopsy was obtained from retroperitoneal nodes that were progressing at that time; the second biopsy was obtained from the lung metastases, the site of progression post-AKT inhibitor treatment. Tumor heterogeneity is a veritable challenge and not only can the molecular profile of cancer change over time, the molecular profile(s) of different metastatic sites can be incongruent^41^. Admittedly, it is possible that different metastatic sites may harbor heterogeneous gene alterations, which could include the possibility of the pre-existence of mTOR A1459D clones in the non-biopsied metastatic site. Another limitation is that the mTOR A1459D mutation was not evaluated functionally in our study for confirmation of the gain of function. However, there are data published which suggest that this is an activating mutation; in addition, another variant in the same site, A1459P, has already been shown to be activating experimentally. We, thus, felt that this alteration was actionable in the context of clinical trial enrollment. This report is significant for both reporting of mTOR as a potential resistance mechanism for AKT inhibition as well as the clinical response to a mTORC1/2 inhibitor post AKT inhibitor resistance.

Our patient demonstrated impressive disease stability with allosteric AKT inhibition for 27 months, and on the progression of disease genomic profiling revealed downstream activating mTOR mutation A1459D, as well as the persistence of AKT1 E17K. Of note, the allosteric inhibitor AKT inhibitor ARQ751, used in this patient inhibits AKT E17K in preclinical models^42^. Treatment with dual mTOR inhibitor sapanisertib (TAK228) in combination with metformin achieved a PR on imaging: potentially even a superior response (PR rather than SD), due to more potent ATP-competitive inhibition, and the downstream inhibition of the two oncogenic lesions. In this trial, metformin was used in combination with sapanisertib as metformin activating AMP-dependent kinase (AMPK) causes phosphorylation and activation of the tumor suppressor gene TSC2, which exerts an inhibitory effect on mTOR^43^; pre-clinically, metformin-induced activation of AMPK has been shown to inhibit cell proliferation, reduce colony formation, and inhibit MAP kinase, AKT, and mTOR^44^, therefore use of metformin in this patient may have also contributed to the enhanced anti-tumor effect.

To our knowledge, we report the first clinical case of acquired resistance following ATP-competitive AKT inhibition due to the development of activating mTOR A1459D, and the first documented response to ATP-competitive mTOR inhibition in this setting. Our case exemplifies precision medicine in action from the ability to rapidly identify a patients’ oncogenic driver, to allow physicians to precisely target drivers of disease in real-time. Furthermore, our case underscores the importance of longitudinal genomic profiling in modern cancer care, to guide management, allowing for the rapid identification of molecular mechanisms of resistance and identifying approaches to overcome resistance.

## Methods

### Participant

The patient was treated with allosteric pan-AKT inhibitor ARQ751 following enrollment to phase I study of ARQ751 (NCT02761694) after the collection of the written informed consent. The patient was subsequently treated with mTORC1/2 inhibitor sapanisertib (TAK-228) following enrollment to a phase I study of sapanisertib (TAK-2280) with metformin 500 mg twice daily (NCT03017833) and collection of written informed consent.

### Materials

Tumor samples were obtained by core biopsy performed by an interventional radiologist. FFPE specimens derived from fresh tumor biopsies were reviewed by an MD Anderson pathologist to ensure adequate tumor cellularity (≥ 20%) for analysis. Tumor samples were evaluated using hematoxylin and eosin staining for tumor cellularity. DNA was extracted, purified, and quantified. All procedures were performed in a CLIA-compliant environment. For genomic analysis, the pre-treatment sample was sequenced and subsequently analysed in the MD Anderson CLIA molecular diagnostic laboratory using the Ion Ampliseq 50-Gene Assay for the detection of mutations in the coding sequence of 50 genes (Thermo Fisher Scientific, MA, USA). DNA extracted from the lung metastasis biopsy after progression on the Akt inhibitor was sequenced along with matched normal DNA from blood, in the MD Anderson CLIA molecular diagnostic laboratory utilizing the Oncomine® platform (Thermo Fisher) for the detection of somatic mutations in the coding sequence of 143 cancer-related genes, as previously described^45^. The pre-treatment DNA sample was sequenced using the Ion Ampliseq 50-Gene Assay and was subsequently re-sequenced on the Oncomine platform to confirm the presence of AKT1 E17K mutation, but not mTOR A1459D mutation. All alterations detected were listed in Supplementary Table 1. The radiologic response was assessed according to RECISTv1.1.

### Reporting Summary

Further information on research design is available in the Nature Research Reporting Summary linked to this article.

## Supplementary information

Reporting Summary

Supplementary Information

## Data Availability

Samples were sequenced and analysed in a CLIA-compliant MD Anderson laboratory as described above. The raw sequencing data are not publicly available due to data privacy regulations and restrictions for use of such data, as stated in the study protocol and patient consent form. The alterations identified on the targeted panels are available in Supplementary Table 1.
