# Supplementary material for: Emergence of mTOR mutation as an acquired resistance mechanism to AKT inhibition, and subsequent response to mTORC1/2 inhibition
Source: NPJ Precis Oncol. 2021 Dec 1;5:99. doi: 10.1038/s41698-021-00240-w (PMC8636467; doi:10.1038/s41698-021-00240-w)
Supplement: Supplementary file 2 — Supplementary Information [file 41698_2021_240_MOESM2_ESM.pdf]

Platform used	Site of tumor biopsy	Functionable Mutation detected	PODS annotation		Co-occurring alteration	PODS annotation	
			Functional significance	Actionable variant		Functional significance	Actionable variant
MD Anderson CMS50 (Ion Ampliseq 50-Gene Assay)	Retroperitoneal lymph node	AKT1 E17K	Activating	Yes	CTNNB1_G34R	No	No
MD Anderson STGA-DNA 2018	Lung	AKT1 E17K	Activating	Yes	CTNNB1_G34R	No	No
		mTOR A1459D	Activating	Yes			
MD Anderson STGA-DNA 2018	Retroperitoneal lymph node	AKT1 E17K			CTNNB1_G34R, SLX4		

Supplementary Table 1: Details of sequential NGS analyses performed.

Abbreviations: PODS, Precision Oncology Decision Support Team
